# Associations of Problematic Internet Use, Weight-Related Self-Stigma, and Nomophobia with Physical Activity: Findings from Mainland China, Taiwan, and Malaysia

**DOI:** 10.3390/ijerph191912135

**Published:** 2022-09-25

**Authors:** Wei Liu, Jung-Sheng Chen, Wan Ying Gan, Wai Chuen Poon, Serene En Hui Tung, Ling Jun Lee, Ping Xu, I-Hua Chen, Mark D. Griffiths, Chung-Ying Lin

**Affiliations:** 1 Chinese Academy of Education Big Data, Qufu Normal University, Qufu 273165, China; 2 Department of Medical Research, E-Da Hospital, Kaohsiung 82445, Taiwan; 3 Department of Nutrition, Faculty of Medicine and Health Sciences, Universiti Putra Malaysia, Serdang 43400, Malaysia; 4 Sunway University Business School, Sunway University, Petaling Jaya 47500, Malaysia; 5 Division of Nutrition and Dietetics, School of Health Sciences, International Medical University, Bukit Jalil, Kuala Lumpur 57000, Malaysia; 6 Department of Educational Psychology, School of Leisure Sports and Management, Guangzhou Sport University, Guangzhou 510500, China; 7 International Gaming Research Unit, Psychology Department, Nottingham Trent University, Nottingham NG1 4FQ, UK; 8 Institute of Allied Health Sciences, College of Medicine, National Cheng Kung University, Tainan 701, Taiwan

**Keywords:** region comparison, nomophobia, physical activity, smartphone addiction, social medial addiction, weight stigma

## Abstract

Insufficient physical activity is a common problem for university students because they may engage in sedentary lifestyle owing to excessive time spent on their smartphones and social media use. This may result in problematic internet use (PIU) and nomophobia (fear of not having a mobile phone). Moreover, prior evidence shows that weight-related self-stigma is an important factor contributing to low physical activity. Therefore, the present study examined the associations between PIU, nomophobia, and physical activity among university students across mainland China, Taiwan, and Malaysia. Participants (3135 mainland Chinese, 600 Taiwanese, and 622 Malaysian) completed the Bergen Social Media Addiction Scale (BSMAS), Smartphone Application-Based Addiction Scale (SABAS), Nomophobia Questionnaire (NMPQ), Weight Self-Stigma Questionnaire (WSSQ), and International Physical Activity Questionnaire Short Form (IPAQ-SF). The measurement invariance of the assessed questionnaires was supported across the three regions. The present findings analyzed using partial least squares structural equation modeling showed that (i) greater nomophobia was associated with higher levels of physical activity, (ii) greater weight-related self-stigma was associated with higher levels of physical activity, and (iii) greater nomophobia was associated with greater weight-related self-stigma. Although the present findings suggest the possibility that experiencing some level of nomophobia or weight-related self-stigma appears to help improve physical activity, it is not recommended that these be encouraged, but reducing PIU should be targeted as a means to improve physical activity.

## 1. Introduction

The World Health Organization (WHO) recommends that, on a weekly basis, all adults should undertake 150–300 min of moderate-intensity, 75–150 min of vigorous-intensity physical activity, or some equivalent combination of moderate-intensity and vigorous-intensity aerobic physical activity [1]. This level of physical activity is recommended because studies have shown that it can (i) prevent excessive weight gain, (ii) help reduce the risk of non-communicable diseases (e.g., type 2 diabetes, cardiovascular disease) and some forms of cancer (e.g., colon, breast), (iii) improve general well-being (e.g., improved sleep quality and reduced depression), and (iv) increase cognitive capacity [1]. Worldwide, one in four adults do not meet the WHO’s global recommendations for physical activity [2]. Given the changing patterns of transportation, increased use of technology, and urbanization, levels of inactivity can be as high as 70% among adults in some countries [2].

Previous longitudinal studies have found an increase in physical inactivity during the transition from adolescence to adulthood and throughout the university years [3,4]. University students from various countries have been found to have low physical activity levels, leading to poor health outcomes [5]. Moreover, the COVID-19 pandemic led to populations in many countries being confined to their homes as a way to inhibit the spread of the virus. Such laws and directives have changed individuals’ lifestyles by reducing physical activity levels while increasing sedentary behavior owing to increased internet use [6]. Consequently, the physical activity of university students should be further explored.

There is increasing recent evidence showing that weight-related self-stigma (or internalized weight stigma) is a factor contributing to physical inactivity among university students [7,8,9]. Weight-related self-stigma has been linked with a wide range of negative physical and mental health outcomes, including depression, anxiety, low self-esteem, poor body image, disordered eating, food addiction, poor health-related quality of life, and avoidance of exercise [10]. Weight-related self-stigma is commonly reported among individuals with higher body weight [11]. When an individual experiences weight-related self-stigma, this may reduce their self-confidence or belief that they have the power and ability to participate in physical activities [12]. This is likely to negatively affect their perceived competence, and subsequently discourage them from doing physical activity, as well as encouraging sedentary behaviors such as internet surfing, online gaming, and excessive use of smartphones [8]. However, Meadows and Bombak [12] suggested that individuals with any weight, size, or shape who wish to engage in physical activity should have no issue to do so in a safe non-stigmatizing space and enjoy doing so with no stress and shame. A study conducted among young adults in Hong Kong found a relationship between weight-related self-stigma and physical activity through perceived behavioral control, and this was especially the case among those who were overweight [11]. Therefore, it is important to examine the association between weight-related self-stigma and physical activity among university students with different body weight status.

Little is known about the association between weight-related self-stigma and exercise outcomes, including motivation. Some research suggests that while both types of weight stigma may be associated with higher levels of introjected motivation (exercising out of a sense of guilt, shame, or obligation) in some individuals, they are not associated with more intrinsic forms of motivation—the types that are likely to lead to ongoing adherence to an exercise regimen and to the optimal psychological and physical benefits accruing from it, or with current physical activity levels [13]. Furthermore, studies have predominantly used the Weight Bias Internalization Scale (WBIS) or the Weight Self-Stigma Questionnaire (WSSQ) to assess weight-related self-stigma [10]. In fact, much of the research on weight stigma and physical activity has been conducted using the WBIS [11,14]. Moreover, there is suggestion that the WBIS assesses the fear of being stigmatized as much as it assesses self-devaluation [15]. There are fewer studies examining weight-related self-stigma assessed with the WSSQ and physical activity outcomes [16]. However, Maïano et al. [16] only used the Fear of Enacted Stigma sub-scale of the WSSQ, and their results showed that it did not significantly predict the involvement in physical activity outside of school among secondary school students with high body weight. Therefore, it is worth assessing weight-related self-stigma using the WSSQ to further determine its association with physical activity among university students.

The issue of weight-related self-stigma and low physical activity is also evidenced by another recent study by Xu et al. [14] during the COVID-19 pandemic. Xu et al. [14] found that weight-related self-stigma could be increased by problematic internet use (typically defined as an excessive use of the internet that compromises individuals’ relationships, occupation, and/or education). During the COVID-19 pandemic, the internet has become an important medium for learning and communication, particularly among university students. There has been a significant surge in social media consumption since the onset of the COVID-19 pandemic [17]. This has led some to claim that this has predisposed university students to be addicted to smartphones and social media [18]. However, accessing social media via smartphones could be an important source of weight-related self-stigma during the COVID-19 pandemic. A longitudinal study conducted by Fung et al. [19] across different epidemic periods of COVID-19 in China showed that social media addiction and smartphone addiction increased perceived weight stigma among schoolchildren. Social media may play a vital role in both exacerbating and lessening weight-related self-stigma owing to its widespread use and ability to link individuals with different experiences [20]. In other words, social media may serve as a platform for body shaming [21] and increased weight-related self-stigma, but it can also serve as a good platform for individuals to receive support and promote positive body image [20]. Moreover, prior evidence has shown that higher levels of problematic internet use are associated with lower levels of physical activity [22]. Therefore, problematic internet use, and more specifically social media addiction and smartphone addiction, should be taken into consideration when investigating the association between weight-related self-stigma and physical activity among university students, especially during the COVID-19 pandemic.

Apart from the association between problematic internet use and weight-related self-stigma, problematic internet use may cause individuals’ fear of not having smartphone (i.e., nomophobia (no-mobile phobia)). Nomophobia is an emerging threat for social, mental, and physical health among young adults [23]. However, inconsistent findings have been shown in the association between nomophobia and physical activity. Xu et al. [14] reported that nomophobia was found to reduce physical activity via weight-related self-stigma and time spent on outdoor activity among female university students in China. In contrast, another recent study in Saudi Arabia by AlMarzooqi et al. [24] did not find any association between nomophobia and physical activity. One potential reason for the contrasting findings is that individuals may use their smartphones for different reasons. When individuals use smartphones as fitness accessories, nomophobia may be positively associated with physical activity. When individuals use smartphones for sedentary activity purposes (e.g., online shopping), nomophobia may be negatively associated with physical activity. Nevertheless, given the rapid development of new technologies and the rising use of numerous types of technological devices such as smartphones, tablets, and laptops among young adults, research that identifies their relationships with physical activity is both vital and timely.

**Figure 1:**
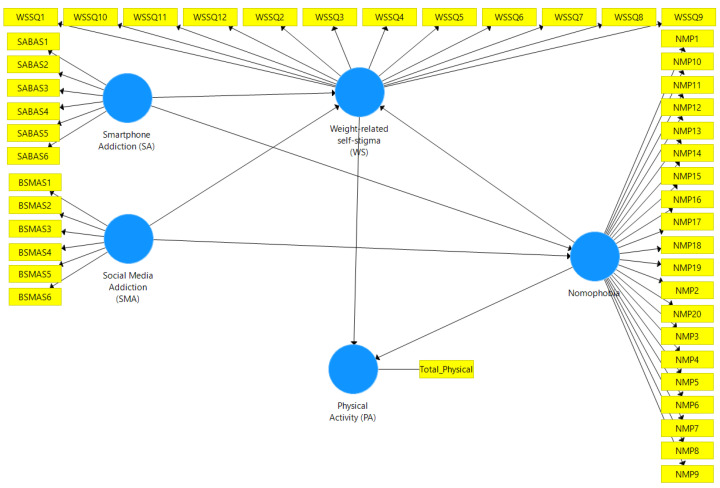
Conceptual model of the study. WSSQ—Weight Self-Stigma Questionnaire; SABAS—Smartphone Application-Based Addiction Scale; NMPQ—Nomophobia Questionnaire. PA was assessed using the International Physical Activity Questionnaire-Short Form.

The present study proposes a conceptual model to investigate the associations between problematic internet use (i.e., social media addiction and smartphone addiction), nomophobia, weight-related self-stigma, and physical activity among university students in mainland China, Taiwan, and Malaysia (Figure 1). It was hypothesized that (i) weight-related self-stigma would be negatively associated with physical activity, (ii) problematic internet use and nomophobia would be positively associated with weight-related self-stigma, and (iii) problematic internet use and nomophobia would be negatively associated with physical activity among university students in the three different regions. There are similarities and cultural differences between mainland China, Taiwan, and Malaysia. In terms of similarity, they are all Asian regions under the influence of collectivism and Confucianism. However, they have different regional cultures, resulting in some different lifestyles (e.g., food tastes are different, physical activity infrastructures and facilities are different). Therefore, testing the associations between problematic internet use, nomophobia, weight-related self-stigma, and physical activity across the three regions could provide useful and bespoke suggestions for the healthcare providers in each region.

## 2. Materials and Methods

### 2.1. Participants, Recruitment, and Ethical Concerns

#### 2.1.1. Mainland China

The Human Experimental Ethics Committee in Guangzhou Sport University (Ref no. 2021LCLL-23) approved the study conducted among mainland Chinese university students. Participants from mainland China were recruited using convenience sampling, where the dissemination of the online survey link (or QR code) was carried out via the *Star* application across 26 Chinese universities. The survey period for mainland Chinese participants was between November and December 2021.

#### 2.1.2. Taiwan

The institutional review board of Chi Mei Medical Center (IRB approval number: 11007-006) approved the study conducted among Taiwanese university students. Participants from Taiwan were recruited using convenience sampling, where the dissemination of the online survey link (or QR code) was carried out via some popular Taiwanese social networking platforms (e.g., *LINE*). The online survey was administered using *Google Forms.* The survey period for Taiwanese participants was between August and September 2021.

#### 2.1.3. Malaysia

The Ethics Committee for Research Involving Human Subjects in Universiti Putra Malaysia (Reference Number: JKEUPM-2021-455) approved the study conducted among Malaysian university students. Participants from Malaysia were recruited using convenience sampling, where the dissemination of the online survey link (or QR code) was carried out via some popular Malaysian social networking platforms (e.g., *WhatsApp, Facebook*). The survey period for Malaysian participants was between August and November 2021.

Participants were recruited in a non-probabilistic way. The inclusion criteria were that participants had to (i) be aged 18 years and above, (ii) study in a university either in an undergraduate or postgraduate program, (iii) have the ability to understand the language used in each region’s survey (i.e., simplified Chinese characters in mainland China, traditional Chinese characters in Taiwan, and English in Malaysia), and (iv) provide informed consent on the survey after reading the survey information.

Kline [25] recommended that the sample size is determined by the model complexity and calculated on the basis of the power of analysis. Grefen et al. [26] suggested a power of 95%, a medium effect size, and an alpha of 0.05. Therefore, the minimum sample size needed in the present study was 119. A total of 600 or more completed surveys were returned for each region. Therefore, the sample size was more than sufficient in the present study. More specifically, a total of 4357 university students from China (*N* = 3135), Taiwan (*N* = 600), and Malaysia (*N* = 622) participated in the present study (Table 1).

### 2.2. Measures

Different language versions of the self-administered questionnaire were used in this study, in which the Chinese (simplified) version was used in China, the Chinese (traditional) version was used in Taiwan, and the English version was used in Malaysia.

#### 2.2.1. Physical Activity

This was assessed using the International Physical Activity Questionnaire-Short Form (IPAQ-SF) [27]. It contains seven items assessing leisure time, domestic and gardening activities, work-related activities, and transport-related activities. The frequency and duration of the three specific types of activity assessed were (i) walking, (ii) moderate intensity activities, and (iii) vigorous intensity activities. The level of physical activity of the participants is measured in metabolic equivalent of task (MET)-minutes per week.

#### 2.2.2. Weight-Related Self-Stigma

This was assessed using the Weight Self Stigma Questionnaire (WSSQ) [28]. It contains 12 items including the self-devaluation subscale and the fear of enacted stigma subscale, rated on a five-point scale rating from 1 (*completely disagree*) to 5 (*completely agree*). A higher score indicates a higher level of weight-related self-stigma. In the present study, the WSSQ showed excellent internal consistency reliability, with Cronbach’s alpha coefficients of 0.944 for overall participants, 0.948 for Chinese participants, 0.928 for Taiwanese participants, and 0.941 for Malaysian participants.

#### 2.2.3. Problematic Internet Use (PIU)

Two different psychometric scales were used to assess PIU: the Smartphone Application-Based Addiction Scale (SABAS) and the Bergen Social Media Addiction Scale (BSMAS). The SABAS was used to assess the risk of developing smartphone addiction within the past week [29]. It has six items rated on a six-point scale from 1 (*Strongly disagree*) to 6 (*Strongly agree*), yielding a total score between 6 and 36. A higher score on the SABAS indicates that an individual is more at risk of addiction to a smartphone application. The Bergen Social Media Addiction Scale (BSMAS) was used to assess the risk of social media addiction within the past week [30]. It has six items rated on a five-point scale ranging from 1 (*very rarely*) to 5 (*very often*), yielding a total score between 6 and 30. A higher score on the BSMAS indicates a greater risk of addiction to social media, and a BSMAS score over 19 indicates an individual is at-risk of developing problematic social media use. In the present study, the SABAS showed good internal consistency reliability, with Cronbach’s alpha coefficients of 0.879 for overall participants, 0.879 for Chinese participants, 0.830 for Taiwanese participants, and 0.881 for Malaysian participants. Similarly, the BSMAS also showed good internal consistency reliability for all participants (Cronbach’s α = 0.882), Chinese participants (Cronbach’s α = 0.886), Taiwanese participants (Cronbach’s α = 0.872), and Malaysian participants (Cronbach’s α = 0.865).

#### 2.2.4. Nomophobia

This was assessed using the Nomophobia Questionnaire (NMP-Q) [31]. It contains 20 items comprising four factors of not being able to communicate, losing connectedness, not being able to access information, and giving up convenience. All of the items are rated using a seven-point scale from 1 (*Strongly disagree*) to 7 (*Strongly agree*), with a total score ranging between 20 and 140. A higher score in the NMQ indicates that an individual experiences more fear of being unable to use mobile phones or the internet. The internal consistency of the NMP-Q in the current sample was excellent (Cronbach’s α = 0.974 for the total sample, 0.978 for Chinese participants, 0.950 for Taiwanese participants, and 0.965 for Malaysian participants).

### 2.3. Data Analysis

Apart from the descriptive statistics on participants’ demographics, the normality of the studied variables was tested before the model evaluation. Multivariate skewness and kurtosis, as suggested by Kline et al. [25], were used and the findings indicated that the data were not multivariate normal (Supplementary Table S1). Adopting the guideline proposed by Kline et al. [25] to assess non-normality for multivariate data, Mardia’s multivariate skewness of ±3 and Mardia’s multivariate kurtosis of ±20 suggested that the data fulfilled the assumption of multivariate normality. In a simulation study by Cain et al. [32], the deviation from normal distribution resulted in a greater chance of type 1 error. Therefore, to analyze the data, the partial least squares structural equation modeling (PLS-SEM) technique was adopted using SmartPLS 3.3.9 [33]. The present study employed PLS-SEM owing to the bootstrapping and prediction-oriented variance-based approach compared with covariance-based structural equation modeling (CB-SEM), which is more confirmation-orientated [34,35]. PLS-SEM was chosen to examine the predictability of exogenous variables (smartphone addiction, social media addiction, and weight stigma) on the endogenous variables (nomophobia and physical activity).

Following the two-stage analytical procedures by Gerbing and Anderson [36], validity and goodness of the measurement model were first tested to evaluate the proposed research model [37]. The cut-off value for satisfactory item factor loadings was above 0.5, average variance extracted (AVE) was above 0.5, and composite reliability (CR) was above 0.70 [38]. In addition, Henseler et al. [39] suggested using the heterotrait–monotrait ratio (HTMT) to assess discriminant validity, where acceptable discriminate validity should have HTMT correlations lower than 0.85. More specifically, HTMT is the average of the heterotrait–heteromethod correlations (i.e., the correlations of indicators across constructs measuring different phenomena) relative to the average of the monotrait–heteromethod correlations (i.e., the correlations of indicators within the same construct).

Following the recommendation by Hair et al. [40], the bootstrapping method of 5000 resampling procedures was applied to determine the level of significance of each indicator of weight. Bootstrapping is a resampling technique that draws a large number of subsamples from the original data (with replacement) and estimates models for each subsample [41]. Regarding the model fit for the structural model, calculation of the standardized root mean square residual (SRMR), an indicator proposed by Hu and Bentler [42], was used. With an SRMR value of less than 0.08, the structural model is considered having good fit [43]. All models in the present study achieved an SRMR value of less than 0.08.

The analysis also utilized Henseler et al.’s [44] measurement invariance of composite models (MICOM) procedure to assess measurement invariance, which involved three steps. The three steps are configural invariance, compositional invariance, and equal composite means and variances, all of which are used to test measurement invariance [44]. Configural invariance is interpreted as equal parameterization and method of estimation. To analyze the configuration invariance, the measurement and structural models, as well as the algorithm for all model estimates, are constructed to be the same for the overall sample as well as in each group sample (i.e., equal questionnaire and equal proposed model for the overall sample and each group sample). Next, the compositional invariance was evaluated using indicator weights. When the indicator weights are equal, the invariance is supported. Moreover, the MICOM procedure with the SmartPLS 3.3.9 program [33] using 5000 permutations was conducted to examine the compositional invariance. With a composite score (i.e., c value) larger than 0.95, the invariance is considered to be supported. Finally, the equality of composite mean values and variance was assessed. The invariance of variances and means are supported when the difference in mean values and the difference in variance ratios are close to 0, and a full measurement invariance is concluded. Nevertheless, if full measurement invariance cannot be satisfied, further analyses (i.e., multigroup comparison) can be conducted under the consideration of partial measurement invariance [44]. More specifically, differences between different locations were estimated using Partial Least Squares-Multigroup Analysis (PLS-MGA) and the Welch–Satterthwait test [38].

## 3. Results

Table 2 shows that all item factor loadings, CR, and AVE are satisfactory. More specifically, all factor loadings, CR, and AVE were above 0.7. In addition, the values of HTMT shown in Table 3 were all below 0.85, indicating the discriminant validity between these studied concepts was supported. In other words, the construct validity of all of the measures analyzed in the present study was supported. With the satisfactory validity, the structural findings of the proposed model were analyzed.

Table 4 summarizes the results from the structural model evaluation for the overall model, Chinese model, Taiwanese model, and Malaysian model. Generally, the structural model had good fit, as indicated by the SRMR values: 0.059 (overall model), 0.061 (mainland Chinese model), 0.076 (Taiwanese model), and 0.060 (Malaysian model). Regarding the structural model findings, all path coefficients were significant in the overall sample and the mainland Chinese sample. The path coefficients from nomophobia to physical activity and from weight-related self-stigma to physical activity were not significant in the Taiwanese and Malaysian samples. The path coefficient from nomophobia to weight-related self-stigma was not significant in the Taiwanese sample. All other path coefficients were significant in both the Taiwanese and Malaysian samples.

The coefficient determinant R^2^ is used to examine a structural model. Each R^2^ in the present study had a high value, as shown in Table 4. The overall model explained 2.1% of the variance in physical activity, 29.6% of the variance in weight-related self-stigma, and 36.8% of the variance in nomophobia. The mainland China model explained 70.2% of the variance in physical activity, 33.2% of the variance in weight-related self-stigma, and 35.5% of the variance in nomophobia. The Taiwan model explained 0.2% of the variance in physical activity, 24.4% of the variance in weight-related self-stigma, and 41.2% of the variance in nomophobia. Lastly, the Malaysia model explained 0.1% of the variance in physical activity, 19% of the variance in weight-related self-stigma, and 32.2% of the variance in nomophobia.

The measurement invariance results indicated that the configural invariance was fulfilled. Table 5 shows that the c values were all above 0.95, which indicated that compositional invariance occurred. However, the results indicated that both equal variance and equal means were not met. Therefore, the multigroup comparisons were conducted with the consideration of partial invariance. Table 6 demonstrates the findings regarding the region comparisons in the hypothesized model. More specifically, the major significant differences in the region comparisons were (i) the path from nomophobia to physical activity, (ii) nomophobia to weight-related self-stigma, and (iii) weight-related self-stigma to physical activity.

## 4. Discussion

The present study proposed a conceptual model to examine the associations between two types of PIU (i.e., smartphone addiction and social media addiction), nomophobia, weight-related self-stigma, and physical activity among university students across three regions: mainland China, Taiwan, and Malaysia. Although the conceptual model was partially supported using the overall sample (i.e., including all participants from mainland China, Taiwan, and Malaysia), different findings were observed across the three region samples. More specifically, nomophobia was a strong factor explaining physical activity for mainland Chinese students (i.e., greater nomophobia was associated with higher levels of physical activity). However, this was not the case for Taiwanese and Malaysian students. Similarly, weight-related self-stigma was a strong factor explaining physical activity for mainland Chinese students (i.e., greater weight-related self-stigma was associated with higher levels of physical activity), and this association was not found among Taiwanese and Malaysian students. Additionally, greater nomophobia was associated with greater weight-related self-stigma among both mainland Chinese and Malaysian students, but not among Taiwanese students.

Nomophobia and physical activity had positively significant associations among mainland Chinese students, but not among Taiwanese and Malaysian students. However, this positive significant association contradicts prior findings that reported that high levels of nomophobia were associated with low physical activity among female university students [14]. Xu et al. [14] further reported that the negative association between nomophobia and physical activity was mediated by time spent on outdoor activities. In other words, if university students maintain their time on outdoor activity engagement, their physical activity might not decrease. However, the present study did not examine the factor of time spent on outdoor activities, and future studies are needed to explore the role of time spent on outdoor activity engagement. It was interesting that nomophobia was associated with higher levels of physical activity. This perhaps could be explained by the increasing use of apps designed for physical activity engagement including both fitness apps and videogames that include physical activity elements. For example, *Zombies, Run!* provides running scenarios and interactive missions to encourage the players to exercise. *Pokémon GO* as an exergame uses the number of daily walking steps as rewards to motivate players to engage in exercise and outdoor activities [45,46]. Individuals who have nomophobia may worry if they do not have their smartphone nearby and are unable to monitor or be rewarded for their physical activity. The increasing use of such apps and games may also explain why increased smartphone use was associated with increased physical activity. Nevertheless, the present study does not provide any empirical evidence to support the postulation, and future studies are warranted to examine this.

Weight-related self-stigma and physical activity had positive significant associations among mainland Chinese students, but not among Taiwanese and Malaysian students. However, the positive significant association contradicts prior findings, which reported that high levels of weight-related self-stigma were associated with low physical activity levels among female university students [9,14]. Moreover, Saffari et al. [9] reported a U-shape relationship between weight-related self-stigma and physical activity when classifying physical activity into three levels: high, moderate, and low. Among the three levels of physical activity, high and low levels of physical activity had greater weight-related self-stigma than moderate levels of physical activity. Therefore, it is possible that weight-related self-stigma may have diverse effects on physical activity engagement. That is, high levels of weight-related self-stigma may reduce an individual’s motivation to exercise because the individual may be trapped in the ‘*why try?*’ effect [47]. Individuals with high levels of self-stigma could have pessimistic attitudes toward their body weight and believe that they are unable to change their weight status [47]. Therefore, the association between weight-related self-stigma and physical activity engagement cannot be concluded. More evidence is required to explore further the potential mechanisms in the relationship between weight-related self-stigma and physical activity engagement.

The positive associations between smartphone addiction, social media addiction, nomophobia, and weight-related self-stigma concur with the evidence accumulated in the existing literature [9,14,19,48]. More specifically, the two types of PIU (i.e., smartphone addiction and social media addiction) increase time spent on smartphone use and social media among university students. When university students spend more time on smartphones and social media, they have a greater likelihood of being exposed to weight stigma information or posts (e.g., hate descriptions on being overweight, so-called ‘*fat-shaming*’) [20], which subsequently increases their weight-related self-stigma. Moreover, when individuals are addicted to smartphone use or social media use, they are likely to be afraid of having no smartphone around and further develop nomophobia and mental health problems [14,22,49,50,51,52]. Therefore, healthcare providers may consider helping university students to reduce PIU in order to prevent further problems regarding their mental health.

There are some limitations in the present study. First, the present study used a cross-sectional study design to examine the conceptual model. Therefore, the causal relationships proposed in the conceptual model cannot be concluded using the present study’s findings. Future studies should thus use a longitudinal design to confirm the causal relationships proposed in the conceptual model outlined here. Second, participants in the present study were recruited using convenience sampling. Therefore, the representativeness of the present sample is not necessarily strong despite the overall large sample size. The present findings thus have restricted ability in generalizability. Third, the data were collected using self-reported questionnaires. Therefore, biases from self-report such as single rater bias and social desirability bias could not be controlled for in the present study. Lastly, the present study did not collect any data regarding the reasons for smartphone use. Given that different purposes of smartphone use may lead to different associations with physical activity, future studies should collect such data to provide further evidence regarding the association between nomophobia and physical activity.

Based on the present findings, there are two implications. First, some level of weight-related self-stigma might help improve physical activity. That is, when an individual feels the shame of being overweight, the individual is likely to engage in physical activity to overcome the weight concern. However, it is important to find a balance and not let weight-related self-stigma jeopardize individuals’ mental health and lead them to becoming inactive. In other words, individuals are likely to feel that physical activity engagement is useless for the management of their body weight if they have very high levels of weight-related self-stigma [9,14,47]. Moreover, the positive association between weight-related self-stigma and physical activity was only found among mainland Chinese university students. Therefore, healthcare providers should monitor weight-related self-stigma level for university students instead of using weight-related self-stigma as a method to improve their physical activity. Second, smartphone addiction and social media addiction were found to have consistently positive associations with higher levels of weight-related self-stigma and nomophobia. Therefore, healthcare providers may want to design appropriate programs that reduce smartphone addiction and social media addiction among university students. Subsequently, university students’ psychological health could be maintained or improved.

## 5. Conclusions

The first hypothesis that weight-related self-stigma was negatively associated with physical activity was not supported. The second hypothesis that PIU and nomophobia were positively associated with weight-related self-stigma was supported. However, the third hypothesis that nomophobia was negatively associated with physical activity was not supported. Overall, these findings indicated that nomophobia and weight-related self-stigma were potential factors improving physical activity among mainland Chinese university students. However, neither nomophobia nor weight-related self-stigma was a significant factor contributing to physical activity among Taiwanese and Malaysian university students. Although the present findings suggest the possibility that experiencing some level of nomophobia or weight-related self-stigma appears to help improve physical activity, it is not recommended that these be encouraged, but reducing PIU should be targeted as a means to improve physical activity. More specifically, nomophobia and weight-related self-stigma are likely to jeopardize individuals’ mental health (e.g., increase psychological distress) [14,22,49,50,51,52]. Moreover, prior evidence shows that higher levels of weight-related self-stigma may decrease individuals’ motivation in physical activity engagement [53]. Therefore, healthcare providers still have to pay attention to the issues of nomophobia and weight-related self-stigma. Accordingly, the present findings indicated that PIU could lead to nomophobia and weight-related self-stigma. Therefore, designing programs for PIU reduction should be a priority among healthcare providers.

## Figures and Tables

**Table 1:** Characteristics of the participants (*N* = 4357).

Characteristics	*N* (%) or M (SD)			
	Overall (*N* = 4357)	China (*N* = 3135)	Taiwan (*N* = 600)	Malaysia (*N* = 622)
Age	20.66 (3.37)	19.65 (2.38)	22.81 (3.75)	23.68 (4.35)
Gender				
Male	1712 (39.3)	1337 (42.6)	209 (34.8)	166 (26.7)
Female	2645 (60.7)	1798 (57.4)	391 (65.2)	456 (73.3)
Education level				
Undergraduate	3901 (89.5)	3023 (96.4)	395 (65.8)	483 (77.7)
Postgraduate	456 (10.5)	112 (3.6)	205 (34.2)	139 (22.3)
Body mass index (BMI)	23.37 (7.07)	23.89 (7.87)	21.98 (3.72)	22.05 (4.48)
Underweight	877 (20.1)	678 (21.6)	86 (14.3)	113 (18.2)
Normal weight	2427 (55.7)	1645 (52.5)	396 (66.0)	386 (62.0)
Overweight	430 (9.9)	243 (7.8)	100 (16.7)	87 (14.0)
Obese	623 (14.3)	569 (18.1)	18 (3.0)	36 (5.8)
Physical activity	4329.72 (3976.54)	5359.25 (4110.28)	2115.74 (1869.39)	2512.58 (3129.87)
Weight stigma	28.45 (10.17)	27.78 (9.64)	30.62 (10.35)	29.74 (12.03)
Smartphone addiction	20.15 (6.26)	19.26 (6.09)	22.80 (5.44)	22.10 (6.67)
Social media addiction	15.39 (4.92)	14.87 (4.70)	15.92 (4.78)	17.51 (5.51)
Nomophobia	78.83 (26.92)	75.56 (26.52)	93.04 (21.83)	81.55 (28.58)

**Table 2:** Indicator loadings, Cronbach’s alpha, composite reliability, and average variance extracted of the models from Chinese, Taiwanese, and Malaysian participants.

Composite/Indicators	Indicator Loading				Composite Reliability				Cronbach’s Alpha				AVE			
	Overall Model	China Model	Taiwan Model	Malaysia Model	Overall Model	China Model	Taiwan Model	Malaysia Model	Overall Model	China Model	Taiwan Model	Malaysia Model	Overall Model	China Model	Taiwan Model	Malaysia Model
Social media addiction					0.910	0.913	0.904	0.899	0.882	0.886	0.872	0.865	0.629	0.636	0.610	0.596
BSMAS1	0.724	0.720	0.718	0.743												
BSMAS2	0.798	0.786	0.824	0.786												
BSMAS3	0.814	0.828	0.794	0.768												
BSMAS4	0.812	0.814	0.807	0.770												
BSMAS5	0.817	0.827	0.796	0.805												
BSMAS6	0.789	0.806	0.742	0.760												
Nomophobia					0.976	0.980	0.954	0.968	0.974	0.978	0.950	0.965	0.671	0.709	0.512	0.604
NMP1	0.784	0.803	0.685	0.720												
NMP2	0.778	0.800	0.683	0.724												
NMP3	0.789	0.799	0.732	0.771												
NMP4	0.806	0.828	0.654	0.749												
NMP5	0.809	0.836	0.642	0.736												
NMP6	0.810	0.845	0.687	0.739												
NMP7	0.815	0.838	0.636	0.760												
NMP8	0.841	0.858	0.670	0.818												
NMP9	0.810	0.827	0.672	0.777												
NMP10	0.810	0.817	0.748	0.817												
NMP11	0.797	0.810	0.688	0.760												
NMP12	0.820	0.826	0.705	0.815												
NMP13	0.843	0.861	0.763	0.797												
NMP14	0.857	0.877	0.766	0.828												
NMP15	0.855	0.883	0.787	0.789												
NMP16	0.848	0.895	0.807	0.785												
NMP17	0.841	0.869	0.791	0.813												
NMP18	0.847	0.877	0.811	0.806												
NMP19	0.782	0.824	0.658	0.749												
NMP20	0.831	0.859	0.682	0.778												
Smartphone addiction					0.909	0.909	0.876	0.910	0.879	0.879	0.830	0.881	0.626	0.626	0.544	0.628
SABAS1	0.682	0.666	0.584	0.711												
SABAS2	0.755	0.747	0.712	0.811												
SABAS3	0.801	0.800	0.700	0.797												
SABAS4	0.821	0.821	0.772	0.824												
SABAS5	0.835	0.843	0.813	0.803												
SABAS6	0.840	0.854	0.817	0.804												
Weight stigma					0.951	0.955	0.939	0.949	0.944	0.948	0.928	0.941	0.620	0.641	0.563	0.610
WSSQ1	0.728	0.731	0.700	0.740												
WSSQ2	0.742	0.728	0.679	0.769												
WSSQ3	0.813	0.808	0.775	0.818												
WSSQ4	0.809	0.815	0.806	0.822												
WSSQ5	0.703	0.728	0.604	0.667												
WSSQ6	0.772	0.770	0.746	0.758												
WSSQ7	0.730	0.753	0.605	0.683												
WSSQ8	0.827	0.851	0.818	0.812												
WSSQ9	0.816	0.834	0.761	0.837												
WSSQ10	0.835	0.836	0.848	0.839												
WSSQ11	0.848	0.866	0.816	0.850												
WSSQ12	0.812	0.866	0.797	0.755												

BSMAS—Bergen Social Media Scale; NMP—Nomophobia Questionnaire; SABAS—Smartphone Application-Based Addiction Scale; WSSQ—Weight Self-Stigma Questionnaire.

**Table 3:** Discriminant validity ^a^.

	Overall Model					Mainland China Model					Taiwan Model					Malaysia Model				
	1	2	3	4	5	1	2	3	4	5	1	2	3	4	5	1	2	3	4	5
1. Nomophobia	--					--					--					--				
2. PA	0.138	--				0.694	--				0.081	--				0.047	--			
3. SA	0.634	0.163	--			0.617	0.651	--			0.699	0.089	--			0.570	0.053	--		
4. SMA	0.513	0.147	0.723	--		0.499	0.590	0.688	--		0.517	0.058	0.747	--		0.554	0.045	0.808	--	
5. WS	0.451	0.110	0.538	0.473	--	0.477	0.757	0.572	0.476	--	0.340	0.030	0.488	0.504	--	0.379	0.071	0.407	0.417	--

^a^—According to the heterotrait–monotrait ratio, when the ratio is below 0.85, the discriminant validity is supported. PA—physical activity; SA—smartphone addiction; SMA—social media addiction; WS—weight-related self-stigma.

**Table 4:** Path coefficients in the overall sample, as well as the mainland Chinese, Taiwanese, and Malaysian samples.

**Overall**	**β**	**Std. Error**	***t*-Value**	**Hypothesis Testing**	***R* ^2^**
Nomophobia -> PA	0.111	0.015	7.334	Contradicted ^a^	0.021 (PA)
Nomophobia -> WS	0.202	0.017	11.648	Supported	0.296 (WS)
SA -> Nomophobia	0.481	0.018	26.460	Supported	0.368 (Nomophobia)
SA -> WS	0.270	0.020	13.395	Supported	
SMA -> Nomophobia	0.173	0.017	9.923	Supported	
SMA -> WS	0.167	0.019	8.964	Supported	
WS -> PA	0.060	0.021	2.778	Contradicted ^a^	
**Mainland China**	**β**	**Std. Error**	***t*-Value**	**Hypothesis Testing**	***R* ^2^**
Nomophobia -> PA	0.448	0.010	43.435	Contradicted ^a^	0.702 (PA)
Nomophobia -> WS	0.216	0.020	10.913	Supported	0.332 (WS)
SA -> Nomophobia	0.461	0.020	23.160	Supported	0.355 (Nomophobia)
SA -> WS	0.312	0.021	14.514	Supported	
SMA -> Nomophobia	0.188	0.021	9.186	Supported	
SMA -> WS	0.150	0.023	6.591	Supported	
WS -> PA	0.529	0.011	48.575	Contradicted ^a^	
**Taiwan**	**β**	**Std. Error**	***t*-Value**	**Hypothesis Testing**	***R* ^2^**
Nomophobia -> PA	−0.035	0.047	0.749	Not Supported	0.002 (PA)
Nomophobia -> WS	0.057	0.047	1.219	Not Supported	0.244 (WS)
SA -> Nomophobia	0.539	0.040	13.430	Supported	0.412 (Nomophobia)
SA -> WS	0.216	0.061	3.548	Supported	
SMA -> Nomophobia	0.146	0.042	3.447	Supported	
SMA -> WS	0.290	0.051	5.655	Supported	
WS -> PA	0.018	0.043	0.414	Not Supported	
**Malaysia**	**β**	**Std. Error**	***t*-Value**	**Hypothesis Testing**	***R* ^2^**
Nomophobia -> PA	0.017	0.039	0.431	Not Supported	0.001 (PA)
Nomophobia -> WS	0.199	0.045	4.390	Supported	0.190 (WS)
SA -> Nomophobia	0.337	0.054	6.202	Supported	0.322 (Nomophobia)
SA -> WS	0.146	0.049	2.964	Supported	
SMA -> Nomophobia	0.278	0.050	5.576	Supported	
SMA -> WS	0.174	0.055	3.175	Supported	
WS -> PA	0.058	0.049	1.168	Not Supported	

^a^—Original hypothesis is negative association; however, the results showed a significantly positive association. PA—physical activity; SA—smartphone addiction; SMA—social media addiction; WS—weight-related self-stigma.

**Table 5:** Measurement invariance of composite models’ (MICOM) results.

**Mainland China vs. Malaysia**	***c* Value**	**CI 95%**	**Compositional Invariance?**
Nomophobia	1.000	[1.000, 1.000]	Yes
PA	1.000	[1.000, 1.000]	Yes
SA	1.000	[1.000, 1.000]	Yes
SMA	1.000	[0.999, 1.000]	Yes
WS	1.000	[1.000, 1.000]	Yes
	**Difference in Mean Value**	**CI 95%**	**Equal Mean Value?**
Nomophobia	−0.233	[−0.073, 0.067]	No
PA	−1.447	[−0.076, 0.067]	No
SA	−0.449	[−0.069, 0.073]	No
SMA	−0.541	[−0.075, 0.073]	No
WS	−0.199	[−0.071, 0.071]	No
	**Difference in Variances Ratio**	**CI 95%**	**Equal Variance?**
Nomophobia	−0.233	[−0.094, 0.100]	No
PA	−1.447	[−0.712, 0.701]	No
SA	−0.449	[−0.091, 0.100]	No
SMA	−0.541	[−0.109, 0.105]	No
WS	−0.199	[−0.096, 0.105]	No
**Mainland China vs. Taiwan**	***c* Value**	**CI 95%**	**Compositional Invariance?**
Nomophobia	1.000	[1.000, 1.000]	Yes
PA	1.000	[1.000, 1.000]	Yes
SA	1.000	[1.000, 1.000]	Yes
SMA	0.999	[0.999, 1.000]	Yes
WS	1.000	[1.000, 1.000]	Yes
	**Difference in Mean Value**	**CI 95%**	**Equal Mean Value?**
Nomophobia	−0.652	[−0.072, 0.070]	No
PA	−1.79	[−0.076, 0.067]	No
SA	−0.56	[−0.073, 0.068]	No
SMA	−0.222	[−0.074, 0.075]	No
WS	−0.293	[−0.078, 0.072]	No
	**Difference in Variances Ratio**	**CI 95%**	**Equal Variance?**
Nomophobia	0.375	[−0.098, 0.103]	No
PA	−5.771	[−0.364, 0.405]	No
SA	0.211	[−0.095, 0.102]	No
SMA	−0.04	[−0.109, 0.116]	No
WS	−0.135	[−0.095, 0.100]	No
**Taiwan vs. Malaysia**	***c* Value**	**CI 95%**	**Compositional Invariance?**
Nomophobia	0.999	[0.999, 1.000]	Yes
PA	1.000	[1.000, 1.000]	Yes
SA	0.999	[0.999, 1.000]	Yes
SMA	1.000	[0.999, 1.000]	Yes
WS	1.000	[0.999, 1.000]	Yes
	**Difference in Mean Value**	**CI 95%**	**Equal Mean Value?**
Nomophobia	0.426	[−0.091, 0.087]	No
PA	−0.153	[−0.093, 0.093]	No
SA	0.117	[−0.097, 0.091]	No
SMA	−0.298	[−0.092, 0.091]	No
WS	0.077	[−0.096, 0.083]	Yes
	**Difference in Variances Ratio**	**CI 95%**	**Equal Variance?**
Nomophobia	−0.520	[−0.113, 0.112]	No
PA	−1.031	[−0.458, 0.444]	No
SA	−0.401	[−0.118, 0.114]	No
SMA	−0.282	[−0.119, 0.122]	No
WS	−0.293	[−0.113, 0.111]	No

*c* value—compositional value, >0.95 indicates invariance. Differences in mean values and variances ratios close to 0 indicate invariance. PA—physical activity; SA—smartphone addiction; SMA—social media addiction; WS—weight-related self-stigma.

**Table 6:** Multi-group analysis test results.

**Mainland China vs. Malaysia.**	**Path Coefficients (China)**	**Path Coefficients (Malaysia)**	**Diff (China–Malaysia)**	***t*-Value**	**Henseler MGA** ***p*-Value**
Nomophobia -> PA	0.448	0.017	0.431	14.620	<0.001
Nomophobia -> WS	0.216	0.199	0.017	0.334	0.751
SA -> Nomophobia	0.461	0.337	0.125	2.539	0.025
SA -> Weight Stigma	0.312	0.146	0.166	3.097	0.001
SMA -> Nomophobia	0.188	0.278	−0.090	1.773	0.082
SMA -> WS	0.150	0.174	−0.023	0.437	0.681
WS -> PA	0.529	0.058	0.471	14.258	<0.001
**Mainland China vs. Taiwan**	**Path Coefficients (China)**	**Path Coefficients (Taiwan)**	**Diff (China–** **Taiwan)**	***t*-Value**	**Henseler MGA** ***p*-value**
Nomophobia -> PA	0.448	−0.035	0.483	15.308	<0.001
Nomophobia -> WS	0.216	0.057	0.160	2.998	0.004
SA -> Nomophobia	0.461	0.539	−0.077	1.573	0.107
SA -> Weight Stigma	0.312	0.216	0.096	1.648	0.153
SMA -> Nomophobia	0.188	0.146	0.042	0.863	0.379
SMA -> WS	0.150	0.290	−0.140	2.490	0.011
WS -> PA	0.529	0.018	0.511	16.136	<0.001
**Taiwan vs. Malaysia**	**Path Coefficients (Taiwan)**	**Path Coefficients (Malaysia)**	**Diff (Taiwan–Malaysia)**	***t*-Value**	**Henseler MGA** ***p*-value**
Nomophobia -> PA	0.017	−0.035	−0.052	0.879	0.380
Nomophobia -> WS	0.199	0.057	−0.143	1.997	0.046
SA -> Nomophobia	0.337	0.539	0.202	3.157	0.001
SA -> Weight Stigma	0.146	0.216	0.070	0.877	0.385
SMA -> Nomophobia	0.278	0.146	−0.132	2.045	0.045
SMA -> WS	0.174	0.290	0.116	1.552	0.114
WS -> PA	0.058	0.018	−0.040	0.593	0.560

PA—physical activity; SA—smartphone addiction; SMA—social media addiction; WS—weight-related self-stigma; Diff—difference; MGA—multigroup analysis.

## Data Availability

The datasets generated during and/or analyzed during the current study are available from the corresponding authors upon reasonable request.

## Supplementary Materials

The following supporting information can be downloaded at https://www.mdpi.com/article/10.3390/ijerph191912135/s1, Table S1: Mardia’s multivariate skewness and kurtosis.

## References

[1] Bull F.C., Al-Ansari S.S., Biddle S., Borodulin K., Buman M.P., Cardon G., Carty C., Chaput J.-P., Chastin S., Chou R. (2020). World Health Organization 2020 guidelines on physical activity and sedentary behaviour. Br. J. Sports. Med..

[2] World Health Organization (WHO) (2018). Global Action Plan on Physical Activity 2018–2030: More Active People for a Healthier World.

[3] Aira T., Vasankari T., Heinonen O.J., Korpelainen R., Kotkajuuri J., Parkkari J., Savonen K., Uusitalo A., Valtonen M., Villberg J. (2021). Physical activity from adolescence to young adulthood: Patterns of change, and their associations with activity domains and sedentary time. Int. J. Behav. Nutr. Phys. Act..

[4] Corder K., Winpenny E., Love R., Brown H.E., White M., Sluijs E.V. (2019). Change in physical activity from adolescence to early adulthood: A systematic review and meta-analysis of longitudinal cohort studies. Br. J. Sports. Med..

[5] Memon A.R., Gupta C.C., Crowther M.E., Ferguson S.A., Tuckwell G.A., Vincent G.E. (2021). Sleep, and physical activity in university students: A systematic review and meta-analysis. Sleep Med. Rev..

[6] López-Valenciano A., Suárez-Iglesias D., Sanchez-Lastra M.A., Ayán C. (2021). Impact of COVID-19 pandemic on university students’ physical activity levels: An early systematic review. Front. Psychol..

[7] Bevan N., O’Brien K.S., Lin C.-Y., Latner J.D., Vandenberg B., Jeanes R., Puhl R.M., Chen I.-H., Moss S., Rush G. (2021). The relationship between weight stigma, physical appearance concerns, and enjoyment and tendency to avoid physical activity and sport. Int. J. Environ. Res. Public Health.

[8] Pearl R.L., Wadden T.A., Jakicic J.M. (2021). Is weight stigma associated with physical activity? A systematic review. Obesity.

[9] Saffari M., Chen J.-S., Wu H.-C., Fung X.C.C., Chang C.-C., Chang Y.-L., Kamolthip R., Potenza M.N., Lin I.-C., Lin C.-Y. (2022). Effects of weight-related self-stigma and smartphone addiction on female university students’ physical activity levels. Int. J. Environ. Res. Public Health.

[10] Pearl R.L., Puhl R.M. (2018). Weight bias internalization and health: A systematic review. Obes. Rev..

[11] Fung X.C.C., Pakpour A.H., Wu Y.-K., Fan C.-W., Lin C.-Y., Tsang H.W.H. (2020). Psychosocial variables related to weight-related self-stigma in physical activity among young adults across weight status. Int. J. Environ. Res. Public Health.

[12] Meadows A., Bombak A.E. (2019). Yes, we can (no, you can’t): Weight stigma, exercise self-efficacy, and active fat identity development. Fat Stud..

[13] Ali Y., Meadows A. Motivations to Exercise: The Good, the Bad, and the Ugly. Proceedings of the 8th Annual International Weight Stigma Conference.

[14] Xu P., Chen J.S., Chang Y.-L., Wang X., Jiang X., Griffiths M.D., Pakpour A., Lin C.-Y. (2022). Gender differences in the associations between physical activity, smartphone use, and weight stigma. Front. Public Health.

[15] Meadows A., Higgs S. (2019). The multifaceted nature of Weight-Related Self-Stigma: Validation of the Two-Factor Weight Bias Internalization Scale (WBIS-2F). Front. Psychol..

[16] Maïano C., Lepage G., Aimé A. (2018). Perceived weight-related victimization and physical activity outcomes among adolescents with overweight and obesity: Indirect role of perceived physical abilities and fear of enacted stigma. Psychol. Sport Exerc..

[17] Cellini N., Canale N., Mioni G., Costa S. (2020). Changes in sleep pattern, sense of time and digital media use during COVID-19 lockdown in Italy. J. Sleep Res..

[18] Islam M.S., Sujan M.S.H., Tasnim R., Mohona R.A., Ferdous M.Z., Kamruzzaman S., Toma T.Y., Sakib M.N., Pinky K.N., Islam M.R. (2021). Problematic smartphone and social media use among Bangladeshi college and university students amid COVID-19: The role of psychological well-being and pandemic related factors. Front. Psychiatry.

[19] Fung X.C.C., Siu A.M.H., Potenza M.N., O’Brien K.S., Latner J.D., Chen C.-Y., Chen I.-H., Lin C.-Y. (2021). Problematic use of internet-related activities and perceived weight stigma in schoolchildren: A longitudinal study across different epidemic periods of COVID-19 in China. Front. Psychiatry.

[20] Clark O., Lee M.M., Jingree M.L., O’Dwyer E., Yue Y., Marrero A., Tamez M., Bhupathiraju S.N., Mattei J. (2021). Weight stigma and social media: Evidence and public health solutions. Front. Nutr..

[21] Eow S.Y., Gan W.Y. (2018). Social media use, body image, and body weight status: Comparison between university students with and without disordered eating in Universiti Putra Malaysia. IJPHCS.

[22] Kwok C., Leung P.Y., Poon K.Y., Fung X.C.C. (2021). The effects of internet gaming and social media use on physical activity, sleep, quality of life, and academic performance among university students in Hong Kong: A preliminary study. Asian J. Soc. Health Behav..

[23] Notara V., Vagka E., Gnardellis C., Lagiou A. (2021). The emerging phenomenon of nomophobia in young adults: A systematic review study. Addict. Health.

[24] AlMarzooqi M.A., Alhaj O.A., Alrasheed M.M., Helmy M., Trabelsi K., Ebrahim A., Hattab S., Jahrami H.A., Saad H.B. (2022). Symptoms of nomophobia, psychological aspects, insomnia and physical activity: A cross-sectional study of esports players in Saudi Arabia. Healthcare.

[25] Kline R.B. (2015). Principles and Practice of Structural Equation Modelling.

[26] Gefen D., Rigdon E.E., Straub D. (2011). An update and extension to SEM guidelines for administrative and social science research. MIS Q..

[27] Craig C.L., Marshall A.L., Sjöström M., Bauman A.E., Booth M.L., Ainsworth B.E., Pratt M., Ekelund U., Yngve A., Sallis J.F. (2003). International physical activity questionnaire: 12-country reliability and validity. Med. Sci. Sports Exerc..

[28] Lillis J., Luoma J.B., Levin M.E., Hayes S.C. (2010). Measuring weight self-stigma: The weight self-stigma questionnaire. Obesity.

[29] Csibi S., Griffiths M.D., Cook B., Demetrovics Z., Szabo A. (2018). The psychometric properties of the smartphone application-based addiction scale (SABAS). Int. J. Ment. Health Addict..

[30] Andreassen C.S., Billieux J., Griffiths M.D., Kuss D.J., Demetrovics Z., Mazzoni E., Pallesen S. (2016). The relationship between addictive use of social media and video games and symptoms of psychiatric disorders: A large-scale cross-sectional study. Psychol. Addict. Behav..

[31] Yildirim C., Correia A.-P. (2015). Exploring the dimensions of nomophobia: Development and validation of a self-reported questionnaire. Comput. Hum. Behav..

[32] Cain M.K., Zhang Z., Yuan K.-H. (2017). Univariate and multivariate skewness and kurtosis for measuring nonnormality: Prevalence, influence and estimation. Behav. Res. Methods.

[33] Ringle C.M., Wende S., Becker J.-M. SmartPLS 3. Bönningstedt: SmartPLS, 2015. http://www.smartpls.com.

[34] Sarstedt M., Ringle C.M., Hair J.F., Homburg C., Klarmann M., Vomberg A.E. (2021). Partial least squares structural equation modeling. Handbook of Market Research.

[35] Sarstedt M., Hair J.F., Pick M., Liengaard B.D., Radomir L., Ringle C.M. (2022). Progress in partial least squares structural equation modeling use in marketing research in the last decade. Psychol. Mark..

[36] Gerbing D.W., Anderson J.C. (1988). An updated paradigm for scale development incorporating unidimensionality and its assessment. J. Mark. Res..

[37] Poon W.C., Tung S.E.H. (2022). The rise of online food delivery culture during the COVID-19 pandemic: An analysis of intention and its associated risk. Eur. J. Manag. Bus. Econ..

[38] Hair J.F., Sarstedt M., Ringle C.M., Gudergan S.P. (2018). Advanced Issues in Partial Least Squares Structural Equation Modeling (PLS-SEM).

[39] Henseler J., Ringle C.M., Sinkovics R.R. (2009). The use of partial least squares path modeling in international marketing. Adv. Int. Mark..

[40] Hair J.F., Babin B.J., Krey N. (2017). Covariance-based structural equation modeling in the Journal of Advertising: Review and recommendations. J. Advert..

[41] Poon W.C., Tung S.E.H. Consumer risk perception of online food delivery during the COVID-19 Movement Control Order (MCO) in Malaysia. J. Foodserv. Bus. Res..

[42] Hu L.T., Bentler P.M. (1999). Cut-off criteria for fit indexes in covariance structure analysis: Conventional criteria versus new alternatives. Struct. Equ. Model..

[43] Henseler J., Ringle C.M., Sarstedt M. (2015). A new criterion for assessing discriminant validity in variance-based structural equation modeling. J. Acad. Mark. Sci..

[44] Henseler J., Ringle C.M., Sarstedt M. (2016). Testing measurement invariance of composites using partial least squares. Int. Mark. Rev..

[45] Farič N., Potts H.W., Rowe S., Beaty T., Hon A., Fisher A. (2021). Running app “Zombies, Run!” users’ engagement with physical activity: A qualitative study. Games Health J..

[46] Zsila A., Orosz G., Bothe B., Toth-Kiraly I., Kiraly O., Griffiths M., Demetrovics Z. (2018). An empirical study on the motivations underlying augmented reality games: The case of Pokémon Go during and after Pokémon fever. Pers. Individ. Differ..

[47] Corrigan P.W., Larson J.E., Rusch N. (2009). Self-stigma and the “why try” effect: Impact on life goals and evidence-based practices. World Psychiatry.

[48] Chen C.Y., Chen I.H., O’Brien K.S., Latner J.D., Lin C.Y. (2021). Psychological distress and internet-related behaviors between schoolchildren with and without overweight during the COVID-19 outbreak. Int. J. Obes..

[49] Daei A., Ashrafi-rizi H., Soleymani M.R. (2019). Nomophobia and health hazards: Smartphone use and addiction among university students. Int. J. Prev. Med..

[50] Oluwole L.O., Obadeji A., Dada M.U. (2021). Surfing over masked distress: Internet addiction and psychological well-being among a population of medical students. Asian J. Soc. Health Behav..

[51] Patel V.K., Chaudhary P., Kumar P., Vasavada D.A., Tiwari D.S. (2021). A study of correlates of social networking site addiction among the undergraduate health professionals. Asian J. Soc. Health Behav..

[52] Ranjan L.K., Gupta P.R., Srivastava M., Gujar N.M. (2021). Problematic internet use and its association with anxiety among undergraduate students. Asian J. Soc. Health Behav..

[53] Puhl R., Suh Y. (2015). Health consequences of weight stigma: Implications for obesity prevention and treatment. Curr. Obes. Rep..

