# Supplementary material for: Associations of Problematic Internet Use, Weight-Related Self-Stigma, and Nomophobia with Physical Activity: Findings from Mainland China, Taiwan, and Malaysia
Source: Int J Environ Res Public Health. 2022 Sep 25;19(19):12135. doi: 10.3390/ijerph191912135 (PMC9566738; doi:10.3390/ijerph191912135)
Supplement: Supplementary file 1 [file ijerph-19-12135-s001.zip › ijerph-1831208-supplementary.pdf]

International Journal of
Environmental Research and Public Health

MDPI

Suppelementary Table S1. Mardia's Multivariate Skewness and Kurtosis.

Variables	β	*p*-value
**Overall model**		
Skewness	34.676	<0.001
Kurtosis	89.470	<0.001
**Mainland China model**		
Skewness	1.765	<0.001
Kurtosis	29.701	<0.001
**Taiwan model**		
Skewness	3.236	<0.001
Kurtosis	30.408	<0.001
**Malaysia model**		
Skewness	13.579	<0.001
Kurtosis	46.996	<0.001
